# Multi-Modal Use of a Socially Directed Call in Bonobos

**DOI:** 10.1371/journal.pone.0084738

**Published:** 2014-01-15

**Authors:** Emilie Genty, Zanna Clay, Catherine Hobaiter, Klaus Zuberbühler

**Affiliations:** 1 Cognitive Science Centre, University of Neuchâtel, Neuchâtel, Switzerland; 2 School of Psychology and Neuroscience, University of St Andrews, Fife, United Kingdom; 3 Living Links, Yerkes National Primate Research Center, Emory University, Atlanta, Georgia, United States of America; CNR, Italy

## Abstract

‘Contest hoots’ are acoustically complex vocalisations produced by adult and subadult male bonobos (*Pan paniscus*). These calls are often directed at specific individuals and regularly combined with gestures and other body signals. The aim of our study was to describe the multi-modal use of this call type and to clarify its communicative and social function. To this end, we observed two large groups of bonobos, which generated a sample of 585 communicative interactions initiated by 10 different males. We found that contest hooting, with or without other associated signals, was produced to challenge and provoke a social reaction in the targeted individual, usually agonistic chase. Interestingly, ‘contest hoots’ were sometimes also used during friendly play. In both contexts, males were highly selective in whom they targeted by preferentially choosing individuals of equal or higher social rank, suggesting that the calls functioned to assert social status. Multi-modal sequences were not more successful in eliciting reactions than contest hoots given alone, but we found a significant difference in the choice of associated gestures between playful and agonistic contexts. During friendly play, contest hoots were significantly more often combined with soft than rough gestures compared to agonistic challenges, while the calls' acoustic structure remained the same. We conclude that contest hoots indicate the signaller's intention to interact socially with important group members, while the gestures provide additional cues concerning the nature of the desired interaction.

## Introduction

A key problem in science is to understand when and how human language evolved and in what aspects it is different from nonhuman animal communication. In terms of timing, one view is that the language faculty emerged ‘de novo’ over the last few million years of hominid evolution, without any relevant precursors. An alternative view is that language emerged more slowly and gradually from older communicative and cognitive skills already present in the primate lineage [1]. One way to address these hypotheses is to look for homologous traits and precursors of human linguistic abilities through the comparative study of primate communication.

In terms of modality, it is unclear whether language evolved from a gestural communication system or whether it has always been based on vocal signals. A relevant finding here is that humans and great apes make frequent use of gestures, while other primates communicate predominantly with vocalisations and facial expressions. Equally relevant is that intentional signalling has been mainly found in great ape gestural communication [2] (but see [3] and [4]), while it is less clear whether vocalisations are also used intentionally. Although primate calls can function to refer to external events, there is usually no strong evidence that they are also produced to deliberately inform a recipient about the event witnessed by the caller [5].

Theories proposing a gestural origin of language suggest that speech was preceded by a gestural phase using visible, voluntarily controlled signals and emphasize the similarities between ape gestural communication and human language [6]. Some empirical work on ape gestures has been on the capacity to convey linguistic content and to communicate with artificial gesture systems, such as American Sign Language [7], [8], [9], [10], [11], [12]. A drawback of these studies is that they have been carried out with captive apes interacting with human caretakers by means of some conditioned behaviour, usually to obtain food. Although interesting, the ecological relevance of these findings has often remained unclear, mainly because great ape natural foraging is not usually based on obtaining or requesting food from social partners. More recent studies have thus focused on gestural communication during natural interactions with conspecifics [13], [14], [15], [16], [17], [18], [19], [20], [21], [22]. These studies have highlighted the flexible use of gestures, in that the same signal is used in a variety of contexts and different signals are used in the same context [23]. There is also some evidence for voluntary control [24], [25], [26] and intentional signalling, that is, signalling in order to alter a recipient's behaviour in a desired way [14], [15], [17], [20], [21], [22], [23], [25] and for the ability to generate novel gestures [18], [19], [20], [23], [27]. These findings, context-independence, voluntary control, intentionality and generativity, are important components of human language, suggesting that they evolved before humans separated from our common ancestor with modern great apes.

Vocal origin of language theories suggest that language derived directly from an earlier communication system, similar to modern primate vocalisations. These theories struggle with the fact that primate vocal communication, in contrast to gestures, is often described as an inflexible, unintentional and involuntary way of reflecting internal states [23]. Partly, this may be because research on primate vocal behaviour has had a different focus, such as whether calls have syntactic organisation [28], [29], referential meaning [30], [31] or whether production is affected by bystanders [4], [32]. A relevant finding is that, in some cases, calls are combined into meaningful sequences [33], [34], indicating that simple rule-based combinations exist in primate vocalisations. The overall consensus is that primate vocalisations can be given to external referents and that listeners can extract information from such calls, but that signallers may not have *intended* to produce them in this way [35]. Another main finding has been that the vocal repertoire of monkeys and apes is highly species-specific and largely inaccessible to vocal learning [36], [37] but see [38]. This is in contrast to call comprehension, which is highly flexible and very responsive to experience [5]. There is also evidence that recipients can infer the intended target of others' vocalisations, even in the absence of visual cues [35].

One problem with the current literature is that there has been little integration between research on gestural and vocal communication [39], [40]. Yet, in natural social interactions, animals regularly produce combinations of acoustic and visual signals and, consequently, studying vocal and gestural communication separately may not be the most fruitful approach to understanding the cognitive underpinnings of animal communication. Although multi-modal signals have been described in various animals during courtship (spiders [41], birds [42]), agonistic interactions (frogs [43]) or anti-predator displays (insects [44], squirrels [45], [46]), primate communication has typically been investigated in separate modalities [40] (but see [47]). However, even in human communication, speech signals are routinely combined with (paralinguistic) vocal and visual signals to convey and modify the speaker's intended meaning [48], [49], [50]. Although there is no doubt that primates regularly produce multi-modal signals, it is currently unknown whether this is merely to increase signal amplitude (i.e. to generate redundancy) or whether it serves a specific semantic function [39]. Experimental studies have shown that chimpanzees (*Pan troglodytes*) combine specific visual, tactile and auditory signals flexibly as a function of the attentional state of a human caretaker [51], [52]. In other studies, Rhesus macaques, *Macaca mulatta*, produced some multi-modal combinations (e.g. screams and facial grimaces) more flexibly than others [53], while in crested macaques, *Macaca nigra*, soft grunts enhanced the effect of lip-smacking by increasing the probability of affiliative contacts [54]. At the neural level, Ghazanfar et al. [55] have identified cells in the auditory cortex of rhesus macaques that are more responsive to bimodal (facial expression and grunts) than uni-modal signals (grunts only), suggesting neurobiological adaptations for multi-modal communication.

In this study, we focus on uni- and multi-modal communication of bonobos (*Pan paniscus*), a close relative of chimpanzees and humans [56]. We systematically investigated a distinct vocal signal, the ‘contest hoot’, which is only given by the males. We were interested in this signal as it is often given as part of multi-modal sequences and directed at other individuals to initiate a social interaction. The exact social function of these calls has remained unclear in the literature. Indeed, according to de Waal [57], p. 206, contest hoots are “…produced by the dominant male to subordinate males and females in the context of aggression”, serve “…as a conspicuous warming up for and warning of an attack or charge”, and are given whilst “…the performer always orients to another individual and gives some form of display, usually a rocking or swaying movement in the same rhythm as the vocalization”. Bermejo & Omedes [58], p. 351 do not use the term ‘contest hoot’, but give a very similar definition to de Waal's [57] as “…peep yelps lengthened into whistles”, highlighting, however, the playful contextual use as “…play-like incitement calls”.

### Aims and predictions

The aims of our study were to describe the use of ‘contest hoot’ in uni- and multi-modal communication, to clarify their functional significance and to assess the structure and meaning of signal sequences. To this end, we first analysed the acoustic structure of contest hoots and how they were combined in multi-modal sequences. We then compared the efficiency of multi-modal sequences with contest hoots given alone, by analysing the recipients' reactions. Judging from the existing literature (e.g. [47], [59]), we predicted that multi-modal sequences were more efficient in triggering responses than contest hoots given alone. We then assessed whether, when used in a socially targeted way, signallers directed contest hoots at specific individuals and whether these targets were strategically selected with regards to their social status. If the signals functioned to assert social status in presence of an audience, we predicted that males preferentially targeted high-ranking individuals that they learnt, from past interactions, were likely to react strongly. Finally, since contest hoots were produced in two very different contexts, agonistic challenge and friendly play, we investigated whether the acoustic structure of contest hoots and the composition of multi-modal sequences differed according to the behavioural context. In line with the general theory that flexibility is larger in primate gestural than vocal signals, we predicted that the call structure would be unaffected by context but that the gesture type would vary to reveal the signaller's intended social goal, i.e., they would selectively produce more rough than soft gestures in the challenge context and conversely in the play context.

## Methods

### Ethics statement

This was a purely observational study that did not contain any interventions. All research adhered to the ethical ASAB/ABS Guidelines for the Use of Animals in Research and was conducted in compliance with animal care regulations and applicable national laws (research permit: MIN.RS/SG/004/2009). We received ethical approval from the scientific coordinator and scientific committee of “Les Amis des Bonobos” (www.friendsofbonobos.org) for this study.

### Study groups

We collected data from two social groups at the ‘Lola ya Bonobo’ sanctuary, Democratic Republic of Congo, between February and June 2012. Both groups live in two large forested enclosures of 10 and 15 ha, respectively, composed of patches of primary rainforest, lakes, swamps, streams, and open grassy areas. In this semi-natural environment, individuals exhibit a large range of behaviours also observed in the wild [60]. During the day, the bonobos can move freely, forage for wild fruits, leaves, and herbaceous vegetation in the forested parts of their enclosures, in addition to three feedings provided by caregivers. The feeding routine is to distribute fruits in the morning, to give a mixture of soya milk (supplemented with milk, maize, honey and nutriments) around midday, and to distribute vegetables in the afternoon. Each day, caregivers distribute approximately 6 kg of fruits and vegetables to each individual. The bonobos are also provided with daily supplemental feeds comprising of seasonal fruits and nuts. Water is freely available from lakes, ponds and streams within their enclosures, with fresh water (with added salt and sugar) additionally distributed several times a week. At night, all individuals are kept in dormitories of approximately 75 m^2^, divided in several separable rooms and connected to the outside enclosures by a tunnel.

### Composition and social dominance hierarchy

During the study period, group 1 consisted of 22 individuals, including adult, subadult and juvenile males and females and 1 infant. Group 2 consisted of 20 individuals with adult, subadult and juvenile males and females, and 1 infant (age classes as defined by [61]). Table S1 shows the group compositions in terms of sex, age class, social status, offspring, and year of arrival at the sanctuary.

We investigated the linearity of the dominance relationships on the basis of matrices of agonistic interactions. ZC collected data on aggression at the time of this study, with fleeing from aggression as a marker for dominance, as demonstrated by previous studies of bonobo social behaviour e.g., [62]. To calculate dominance relationships, we used the Matman analysis programme (Noldus, version 1.1; Wageningen, The Netherlands). Following earlier studies, e.g., [62], [63], we investigated whether the dominance hierarchy was linear by calculating the adjusted linearity index h', which takes into account the number of unknown relationships [63], [64].

### Data collection and analysis

Observations took place over 68 days and included 222 hours of observation time, split equally between the two groups. Observations usually started around 08.30am and continued through mid-afternoon. As all the observations were done in association with feeding times, all members of the group were visible or present at the edge of the forest. Behavioural data were collected using all-occurrence sampling [65] with a focus on how social interactions were initiated and communication behaviour was deployed.

For subsequent analysis, we only considered events that contained contest hoots, either alone or in combination with other signals. Sequences were defined as strings of two or more signals made by the same individual within less than 1 s of each other. Multi-modal sequences were defined as a combination of two or more signals of different sensory modalities (i.e. call and gesture) produced within less than 1 s of each other. If inter-signal intervals surpassed 1 s, we considered them as belonging to separate sequences. This criterion has been used in gestural research and we thus decided to apply it to make our study comparable with previous work [52], [66], [67]. Strings of two or more sequences by the same individual were defined as a communicative bout (as per [67]).

We used Filemaker Pro to administer the resulting database. Social interactions were recorded with a Panasonic HD digital camcorder (HDC-SD900) equipped with a directional microphone (Sennheiser MKE 400).

### Communicative repertoire

We were interested in how contest hoots were combined with gestures, body signals (postures and movements), and facial expressions. To this end, we relied on communicative signals already defined in previous studies with bonobos [7], [19], [47], [57], [68], [69], [70], [71], [72] and other great apes [21], [22]. Table 1 summarises definitions of all non-vocal signals used in combination with contest hoots. In practice, facial expressions were generally difficult to detect consistently and were therefore not further considered in this analysis, which is restricted to combinations of vocalizations, gestures, and body postures and movements. When observable, the most common facial expression associated with contest hoots was ‘silent-teeth baring’ [57].

**Table 1:** List and definition of gestures and body signals used in multi-modal sequences with contest hoots.

Rough Signals	
*Gestures*	
Arm swing (S)	Swinging arm back and forth on side, either once or repetitively
Arm swing with object (S)	Swinging arm back and forth on side, either once or repetitively with object held in hand
Flap (S)	Raising one arm and hand and making a downward slapping movement of the arm in front of recipient
Flap with object (S)	Raising one arm and hand and making a downward slapping movement of the arm in front of recipient with object held in hand
Hit with object (C)	Hitting another individual with object held in hand
Hit ground with object (A)	Hitting ground with object held in hand
Kick (C)	Kicking another individual with foot
Object shake (S)	Shaking fixed object forcefully with one or both hands
Push (C)	Pushing away gently another individual with hand or arm
Rap object (A)	Rapping object on the ground back and forth repetitively
Rhythmic stomp (A)	Stamping the ground alternatively with one foot then the other very rapidly
Slap other (C)	Slapping forcefully and singly another individual with palm of hand
Slap object (A)	Slapping forcefully and singly object with palm of hand
Stomp (A)	Stamping the ground forcefully with sole of foot
Throw object (S)	Throwing an object in direction of another individual
*Body signals*	
Bipedal swagger (S)	Lateral swaying of the upper body
Object dragging (A)	Dragging object held in hand along side of the body (usually branch) while moving forward, charging display
Push object (A)	Pushing away forcefully an object with hand usually with body hunched over andaccompanying a charging display
Stiff trot (S)	Running with stiff forelegs
**Soft signals**	
*Gestures*	
Arm raise (S)	Raising one arm above the head
Arm raise with object (S)	Raising one arm above the head while holding object
Grab (C)	Grabbing gently another individual's body part with closed hand
Grab-pull (C)	Grabbing gently another individual's body part with closed hand and pulling towards self
Hand wave off (S)	Raising arm and waving it away from self
Hand-down reach (S)	Holding a hand toward another individual by extending the arm and hand, palm is facing downwards
Hand-side reach (S)	Holding a hand toward another individual by extending the arm and hand, palm is facing sideways
Hand-up reach (S)	Holding a hand toward another individual by extending the arm and hand, palm is facing upwards
Stretch over (S)	Stretching and raising arm till about head level with the palm facing downwards, sexual invitation
Touch (C)	Touching gently another individual's body part with palm of hand
Wrist shake (S)	Shaking hand vigorously with flexible wrist towards another individual
*Body signals*	
Bipedal present (S)	Standing bipedally in front of recipient with arms spread apart, sexual invitation
Concave back present (S)	Exposing genitals with legs spread wide apart while sitting in front of recipient, sexual invitation
Rump present (S)	Presenting hindquarters while standing quadrupedally in front of recipient, sexual invitation

The table is divided between rough and soft signals, gestures and body signals. Signal sensory channel; A: audible, C: contact and S: silent signals.

We define a gesture as a mechanically ineffective physical movement of the limbs or head, directed towards a recipient and used in a ‘goal-directed’ way to influence its behaviour [22], [73]. Body signals are defined in similar terms for physical movements or postures of the whole body (that can be part of the species typical repertoire such as sexual invitation postures or display behaviours) (table 1). To qualify as ‘goal-directed’, a gesture or body signal has to be accompanied by (a) audience checking (signaller looks at recipient before or during gesturing), (b) response waiting (signaller pauses and maintains visual contact with recipient after gesturing) or (c) persistence and/or elaboration (following response waiting, signaller repeats same signal or uses new signal or combination of signals) [21], [22].

For each gesture and body signal, we determined the sensory modality as ‘silent’, ‘audible’ or ‘tactile’ and the mode of delivery as ‘rough’ or ‘soft’. ‘Rough’ signals were either part of display behaviours (i.e. bipedal swagger, object dragging; see [57], [58], performed with force (i.e. flap) or physically invasive (i.e. slap other). ‘Soft’ signals were silent signals performed without force (i.e. hand reach) and soft contact gestures (i.e. touch; table 1).

### Social interactions

For each interaction containing contest hoots, we coded the (a) identity, sex and age class of signaller and recipient (as identified by the orientation of the signaller), (b) context (agonistic, challenge, affiliative, play, rest, travel, food), (c) recipient's attentional state (fully attending, head direction 45° to 90° from signaller, or not attending), (d) duration of individual contest hoot (s), (e) distance between signaller and recipient (m), (f) duration of multi-modal sequences (s), (g) type of gestures and body signals combined with contest hoots, (h) sensory channel of non-vocal signals (silent, auditory, contact), (i) presence or absence of response waiting, (j) recipient reaction, (k) presence or absence of persistence (repetition of signal and/or elaboration), and (l) success or failure of the interaction.

### Recipient responses

Contest hoots were performed in two different contexts, agonistic challenge or play. They tend to provoke a noticeable reaction in the recipient, although this depended on the context. We classified recipient reactions as ‘weak’, ‘moderate’, or ‘strong’. In both challenge and play interactions, weak reactions included staring at the signaller or avoiding physical contact (by fending oneself or changing body posture). Moderate reactions included stopping a current activity, approaching the signaller, gesturing, vocalising, or moving away. Strong reactions in the challenge context consisted of charging or chasing the signaller with or without vocalisations, typically barks (female recipients, Video S1) or conflicts with minimal physical contact (male recipient, Video S2). None of these reactions ever led to severe aggression. Strong reactions in the play context consisted of mutual play with physical contact (male and female recipients,Video S3). Following a strong reaction (agonistic chase, charge, or play), signallers never made further attempts to interact with the target, suggesting that the desired goal had been met.

### Statistical analysis

Using all-occurrence sampling [65] we focused on all initiations of communicative interactions between two individuals. As a result, not all individuals contributed equally to the final data set. We thus calculated relative frequencies for all individuals, which enabled us to treat the individual as an independent unit. Statistical analyses were carried out with SPSS v11 (*α* level = 0.05). Following Hobaiter & Byrne's [22], [67] protocol, data were checked for their appropriateness for parametric statistics (skew and homogeneity of variance) and, if necessary, we applied appropriate transformations (see Methods S1). If planned comparisons could be made, we used standard *t*-tests or their nonparametric equivalents, with Bonferroni corrections applied. For multiple small data sets, we used replicated G-test for goodness-of-fit (as an alternative to the chi-square test) to check whether each of the smaller data sets fits the expected ratios, i.e. whether all small data sets have a similar pattern of use. In such cases we pooled the data to achieve greater power.

### Acoustic morphology and analyses

Quantitative analyses of the acoustic structure of contest hoots were conducted using Raven Pro 1.4. The contest hoots were analysed using the following spectrogram settings: pitch range: 500–5,000 Hz, spectrogram view range: 0–5 kHz (window length of 0.02 s, dynamic range 70dB). All spectral measurements were taken from the fundamental frequency (F0) (for details on acoustic analysis parameters, see Methods S1 and Figure S1).

We conducted a discriminant function analysis (DFA) to assess whether each of the uncorrelated acoustic variables, when combined in one model, could discriminate between the two contexts in which contest hoots were produced (challenge and play). Each of the 10 males equally contributed five calls (*N* = 50) in the challenge context, but due to small sample sizes and quality of some recordings the males did not contribute equally to the play context. Indeed, out of the seven males that produced contest hoots in the play context, only four contributed five calls, the three others contributed three, two and one calls respectively (*N* = 26).

### Sample size

We collected a total of 523 video clips that contained contest hoots performed by *N* = 7 subadult and *N* = 3 adult males. 47.8% of the clips (*N* = 250) were excluded because (a) significant parts of the interaction were not visible (*N* = 35; 6.7%), (b) calls were only partially audible (*N* = 35; 6.7%), (c) the recipient of the call could not be determined (*N* = 59; 11.3% e.g., in triadic interactions), (d) the calls were not used in a socially directed manner or were directed at a keeper (*N* = 121; 23.1%). The majority of these undirected or keeper-directed calls (*N* = 85) were produced by two individuals (Api and Keza, table S1) during food distribution. In the remaining *N* = 263 clips, we identified 585 socially directed contest hoots (range: 13–138 per male; table 2), for which we coded the variables as described before.

**Table 2:** Individual frequency of contest hoots in the challenge and play contexts for each signaller of group 1 and 2.

Study group	Signallers	Age class	Social status	N contest hoots	
				Challenge (*N* = 460)	Play (*N* = 125)
1	Manono	A	α	73	0
1	Kikwit	A	I	13	0
1	Fizi	SA	α	38	0
1	Lomami	SA	I	103	35
1	Api	SA	I	82	54
1	Matadi	SA	I	9	4
1	Dilolo	SA	I	37	10
2	Keza	A	H	79	6
2	Mbandaka	SA	α	10	14
2	Ilebo	SA	I	16	2

Age classes; A: adult, SA: subadult. Social status; α: alpha male; H: high-ranking; I: intermediate-ranking; L: low-ranking.

### Inter-observer reliability

All data were collected and coded from video clips by EG. To assess inter-observer reliability, 10% of the video clips were recoded by ZC to calculate the accuracy of determining (a) the identity of the signaller and recipient, (b) the type of vocalizations produced by the signaller, (c) the recipient's reaction, (d) the signaller's potential desired goal, and (e) whether or not the signaller was successful in provoking the desired reaction. A sample of 150 vocalisations, including 120 contest hoots and 30 other calls, were also recoded by ZC to assess the inter-observer reliability of call classification.

## Results and Discussion

### Inter-observer reliability

Inter-observer reliability was excellent (video coding: *k* = 0.89 overall, perfect concordance for signaller and recipient identities, type of vocalisation, and recipient's reaction; call classification: *k* = 0.97).

### Uni- and multi-modal use of contest hoots

#### Description of contest hoots

**Figure 1:**
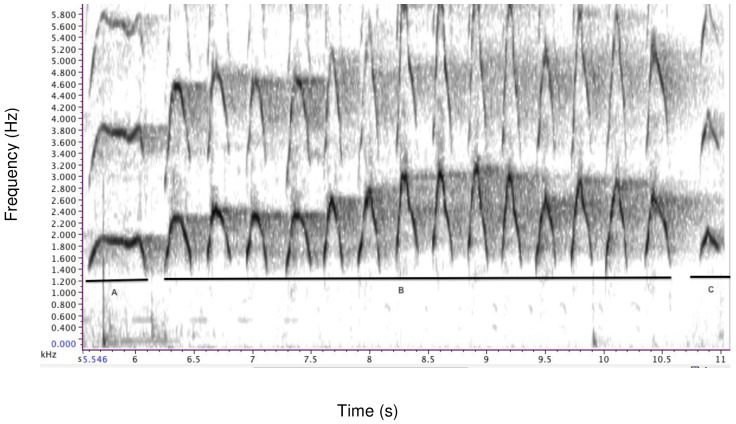
Representative spectrographic illustration of a contest hoot performed by Fizi. The acoustic structure is composed of A: introductory phase, B: escalation phase with *N* = 14 stereotyped units and C: let-down phase.

Contest hoots are call sequences consisting of an introductory phase (modulated inverted u-shape form), an escalation phase composed of several stereotyped units (unmodulated inverted u-shape), and a let-down phase (Figure 1). The composition of the sequence varied with the caller's age. Subadults generally repeated the introductory phase or added one or more stereotyped units of the escalation phase to the introductory phase, but they rarely reach the full escalation and let-down phase. In contrast, adult males usually produced calls with an introductory and escalation phase, composed of several stereotyped units, followed by an occasional let-down phase.

#### Effectiveness of uni- versus multi-modal contest hoots

**Figure 2:**
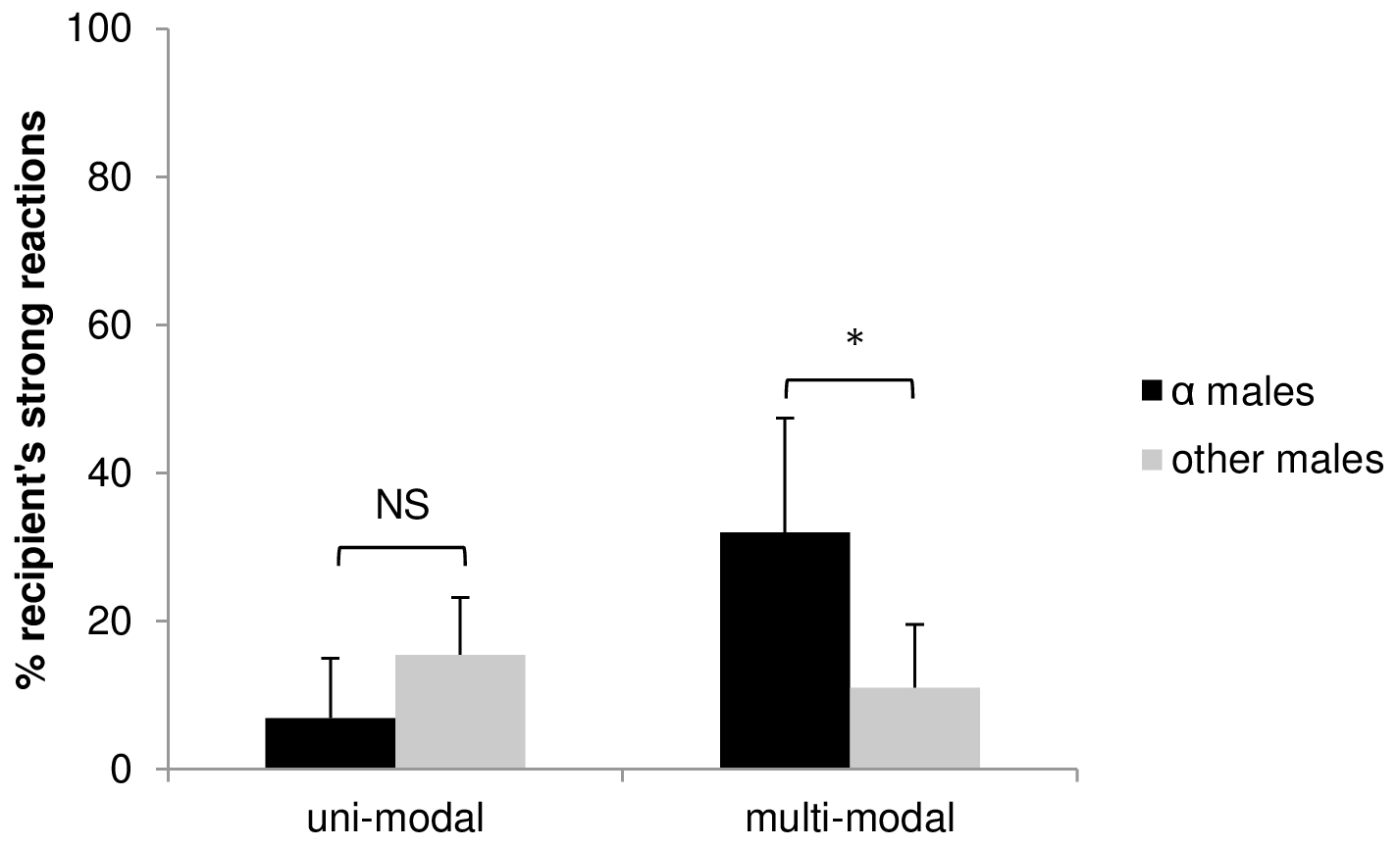
Percentage of strong reactions elicited by uni- and multi-modal contest hoots. Black bars: alpha male (α) signallers, grey bars: other male signallers. NS: non-significant, *P<0.05.

The effectiveness of communicative signals is measured by their propensity to alter the recipient's behaviour and elicit a social reaction. In our sample, we found that, across signallers, multi-modal sequences were not more successful in eliciting reactions in targeted individuals than contest hoots given alone (uni-modal: 80.7±22.1%; multi-modal: 89.2±11.4%, means ± SE; *N* = 10 males; *t* = 1.412, *df* = 9, *P* = 0.191, matched pair *t*-test, two-tailed). The same was the case when analysing strong reactions only (uni-modal: 12.9±8.5%; multi-modal: 17.3±14.3%; means ± SE; *N* = 10 males; *t* = 0.837, *df* = 9, *P* = 0.424; matched pair *t*-test, two-tailed). However, when analysing the three alpha males separately (alpha position changed once within group 1), they were significantly more likely to get strong reactions to multi-modal sequences compared to other males (alpha males: 32.0±15.4%, other males: 11.0±8.5%, means ± SE; *N* = 10; *t* = 2.78, *df* = 8, *P* = 0.024; *t*-test, two-tailed, Figure 2). When analysing contest hoots alone, we found no such difference (alpha males: 6.9±8.1%, other males: 15.4±7.8% means ± SE; *N* = 10; *t* = 1.54, *df*  = 8, *P* = 0.163; *t*-test, two-tailed, Figure 2).

Why were multi-modal sequences of alpha males more likely to cause strong reactions than those of other males? One simple explanation is that the alpha male was generally perceived as more dangerous, thus eliciting stronger responses than other males. A more complex interpretation is that alpha males have experienced more interactions compared to other individuals, and have progressively learned which combinations of signals are most efficient to trigger reactions. In chimpanzees, similar arguments have been made in that older individuals were more likely to use single successful gestures than gesture sequences to communicate, while younger individuals were more likely to use sequences although these were less successful than single gestures [67]. Here, the interpretation was that young individuals did not understand the differences in efficacy so that, by using gesture sequences, they were able to increase the chances of using at least one successful gesture to obtain a response. To test whether bonobos purposefully combine gestures with contest hoots as a function of prior experience of success, it would be necessary to establish the success rates of the gestures when used uni-modally.

### Functional significance

#### Age/sex class of recipient

The distribution of recipients differed significantly across age/sex classes (total G-value = 569.26; pooled G-value = 223.3, *P*<0.001; replicated G-test of goodness of fit) with subadult males and adult females targeted more often than adult males and subadult females (subadult males: *N* = 9, 53.5%; adult males: *N* = 3, 11.1%; subadult females: *N* = 4, 2.7%; adult females: *N* = 9, 30.9%), while the remaining age/sex classes were rarely or never targeted (juvenile males: *N* = 2, 1.5%; infant male: *N* = 1, 0.2%).

#### Preferred targets

Each male signaller had one to four preferred individual target individuals (mean ± SD = 2.70±1.06; table S2) that were selected significantly more often than chance (binomial tests, table S2). Preferred targets differed significantly between males (heterogeneity G-value = 345.96, *P*<0.001; replicated goodness of fit G-test), who were highly selective in whom they targeted (*P*<0.001, goodness of fit tests, for individual results see Table S2).

To assess the effect of social dominance we first determined the dominance hierarchies in both groups, which were linear (group 1: matrix total = 423; h' = 0.50; χ^2^ = 71.04; *df* = 25.84; *P*<0.001; unknown relationships: *N* = 64, 37.4%; one-way relationships: *N* = 100, 58.5%; two-way relationships: *N* = 7, 4.1%; tied relationships: *N* = 2, 1.2%. Group 2: matrix total = 437; h' = 0.50; χ^2^ = 57.21; *df* = 24.14; *P*<0.001; unknown relationships: *N* = 22, 40.0%; one-way relationships: *N* = 31, 56.4%; two-way relationships: *N* = 2, 3.6%; tied relationships: *N* = 1, 1.8%). We then divided each group into three rank clusters at equal points along the list (high-, intermediate- and low-ranking), which corresponded well to our subjective impressions of dominance relationships.

**Figure 3:**
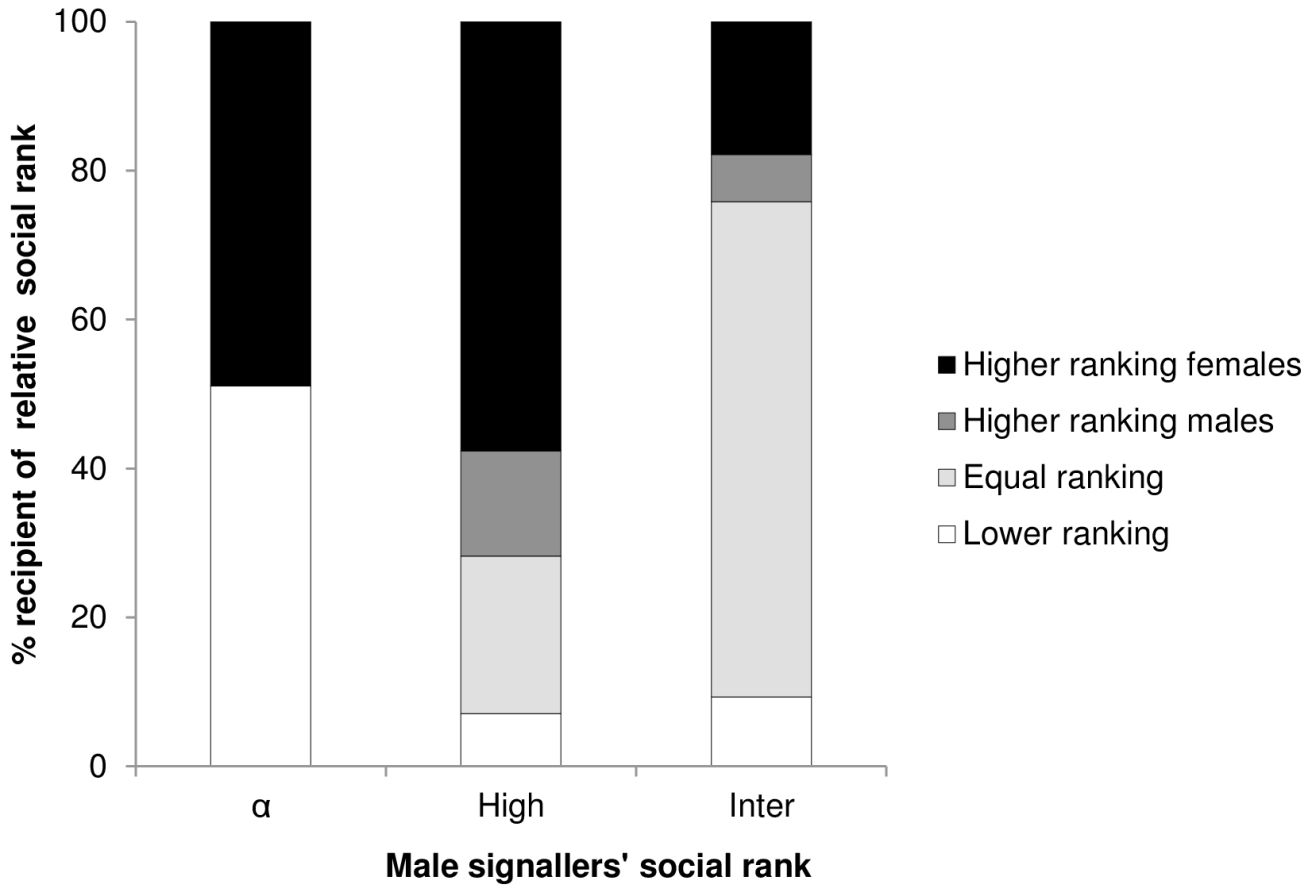
Percentage of contest hoots given by male signallers towards recipients of different relative social rank. Signaller's rank are represented as alpha (α), high and intermediate. Recipients' ranks were calculated relative to the signaller (higher ranking females and males, equal and lower ranking males and females).

We found no differences in the propensity of males (alpha males excluded) to give contest hoots to higher or equal ranking targets (higher ranking: 35.2±31.7%; equal ranking: 51.6±32.2%; means ± SE; *N* = 7; *t*-test, two-tailed: *t* = 0.708, *df* = 6, *P* = 0.506), but a significant difference between higher/equal and lower ranking ones (higher/equal ranking: 43.38±8.73%; lower ranking: 12.9±16.7%; means ± SE; *N* = 7; *t*-test, two-tailed: *t* = 3.163, *df* = 6, *P* = 0.019; Figure3; one exception was observed in the play context, Table S2).

We also found that the alpha males were the only individuals to preferentially target the alpha females (with one exception, Table S2). If they targeted lower ranking males, then they were only individuals who held at least an intermediate rank (Figure 3, Table S2).

These combined results indicate that contest hoots can be produced uni- and multi-modally and in a socially targeted way. Indeed, despite the fact that the effectiveness of auditory signals is less constrained by spatial proximity, the signallers started communicating when at a short distance from their recipient, suggesting that signallers targeted specific individuals with their communication attempts (see Results S1). These targets were mostly subadult males and adult females of equal or higher rank relative to the signaller's.

In bonobo society, females are overall more dominant than males but their dominance is not exclusive [74] in that they are more likely to induce submissive behaviour from high-ranking males when allies are present [75]. We found that the alpha females of each group were the preferential targets of the respective alpha males (with the exception of one subadult male). It may be that, in choosing so, the alpha males sought to demonstrate their high status to others. However, after a change in the male alpha position in group 1, the new alpha male did not immediately start to challenge the alpha female, while the former alpha male continued to do so, suggesting that additional factors may play a role, or that the change in hierarchy was too recent to witness a shift in the preferential selection of targets. All other males preferentially targeted males of equal or higher rank in the challenge context, and if they preferentially targeted lower ranking ones it was only in the context of play.

#### Recipient responses

One way to determine the function of a communication signal is to monitor the behavioural responses of recipients and whether or not the signaller appeared to be satisfied with the response. Strong reactions were charging or chasing the signaller (challenge context) or playing (play context). We did not find any signs of persistence following these reactions, suggesting that the signaller's goal had been met.

**Figure 4:**
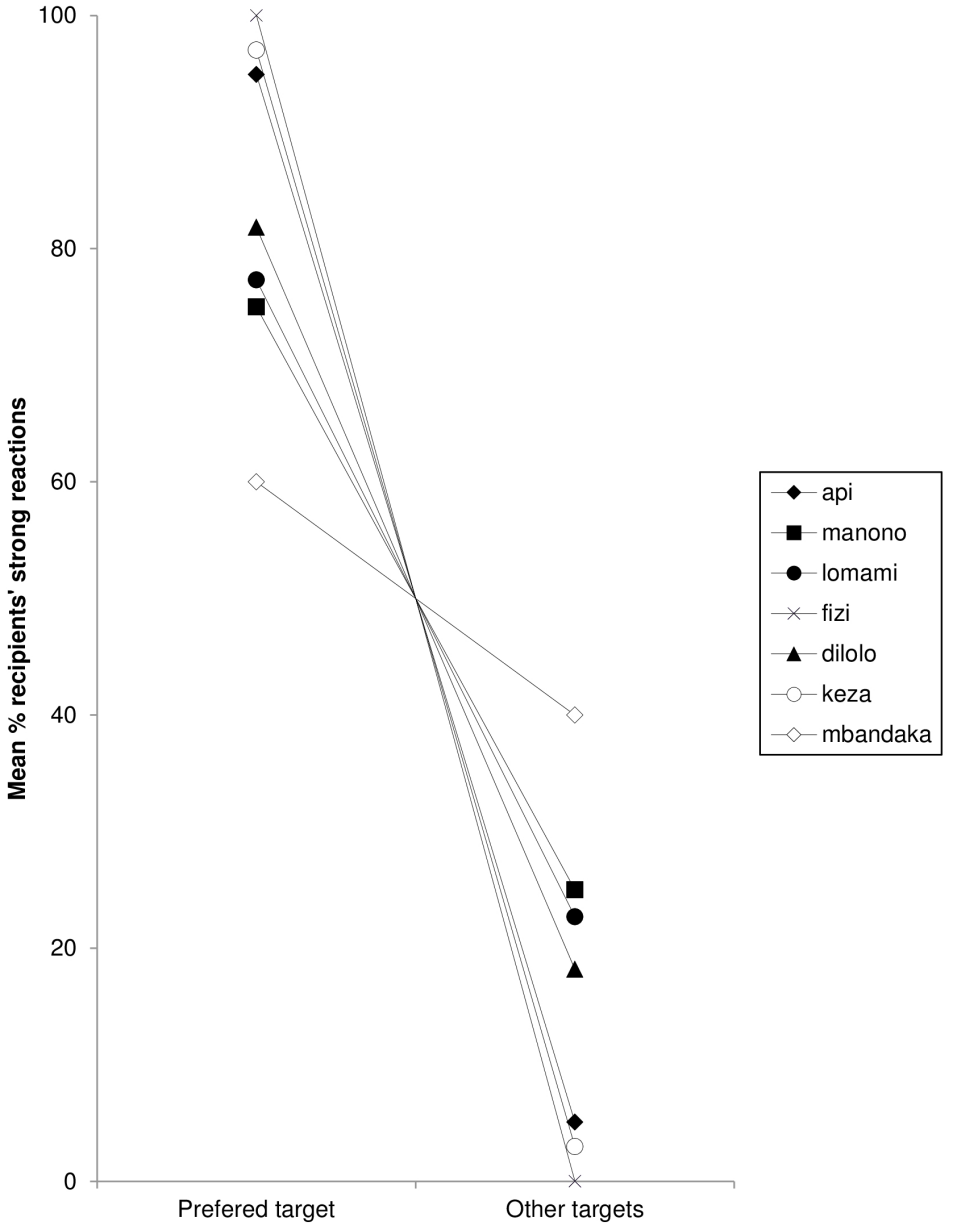
Mean percentage of strong reactions elicited from preferred and all other targets for each signaller. Only seven males participated to the data set, the 3 others elicited no or too few strong reactions.

Recipients reacted by producing observable responses to contest hoot sequences in 80.6% of cases (472 of 585; means ± SE: 84.5±13%). When comparing strong reactions only, preferred targets reacted significantly more often than non-preferred targets (preferred targets: 33±11.7%; other targets: 6.5±7.6%; means ± SE; *N* = 7; paired *t*-test, two-tailed: *t* = 3.866, *df* = 7, *P* = 0.006; for individual differences see Figure 4).

These results indicate that males preferentially targeted individuals that were more likely to react strongly compared to others.

Apart from charging or chasing, we never observed severe aggression or violence following contest hoots production. Males only targeted individuals of higher or equivalent rank relative to their own, and that were more likely to react strongly, with the apparent desire to elicit an agonistic chase. We thus concluded that contest hoots function as a display to assert social status. Since the behaviour was usually done in the presence of an audience, we also concluded that an additional function is to demonstrate to others the ability to provoke an important group member. In sum, contest hoots appear to function as a non-risky way to display one's own and probe others' social ranks in the presence of an audience. There is a growing literature showing that, like humans, animals base decisions about cooperation and competition on the perceived ‘reputation’ of others, acquired through experiences in direct interactions or observations of third-party interactions [76], [77]. Whether or not contest hoots primarily function in reputation formation should be tested more directly in future research.

### Structure and meaning

#### Production context

All subadult and adult males produced contest hoots to challenge others (*N* = 460 events; means ± SE: 80.6±19.6%; table 2), but only one of three adult males produced contest hoots during play, while six subadult males produced the calls in this context (*N* = 125 events; means ± SE: 27.7±17.5%; Table 2).

**Figure 5:**
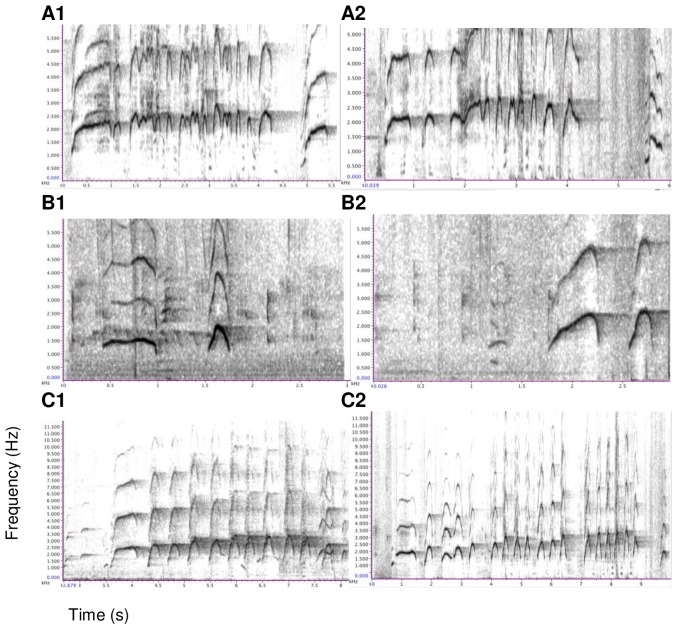
Spectrographic illustrations of contest hoot calls produced during the challenge (1) and play (2) contexts. Calls were produced by three subadult males; A: Api; B: Dilolo; C: Lomami.

The calls produced in both contexts were acoustically indistinguishable (see Figure 5 for individual spectrograms in the two contexts). Following checks for multi-colinearity and singularity, we used 17 of 18 parameters (see Methods S1; ‘duration of escalation’ excluded, *N* = 65 calls) to calculate discriminant functions, one of which significantly discriminated between calls given in the two contexts (Wilks' lambda = 0.638,χ^2^ = 25.17, *df*  = 14, *P* = 0.033). In a cross-validated analysis, it was not possible to successfully classify calls according to context (correct classification: 40/65; binomial test: *P* = 0.08).

#### Multi-modal sequences structure

**Figure 6:**
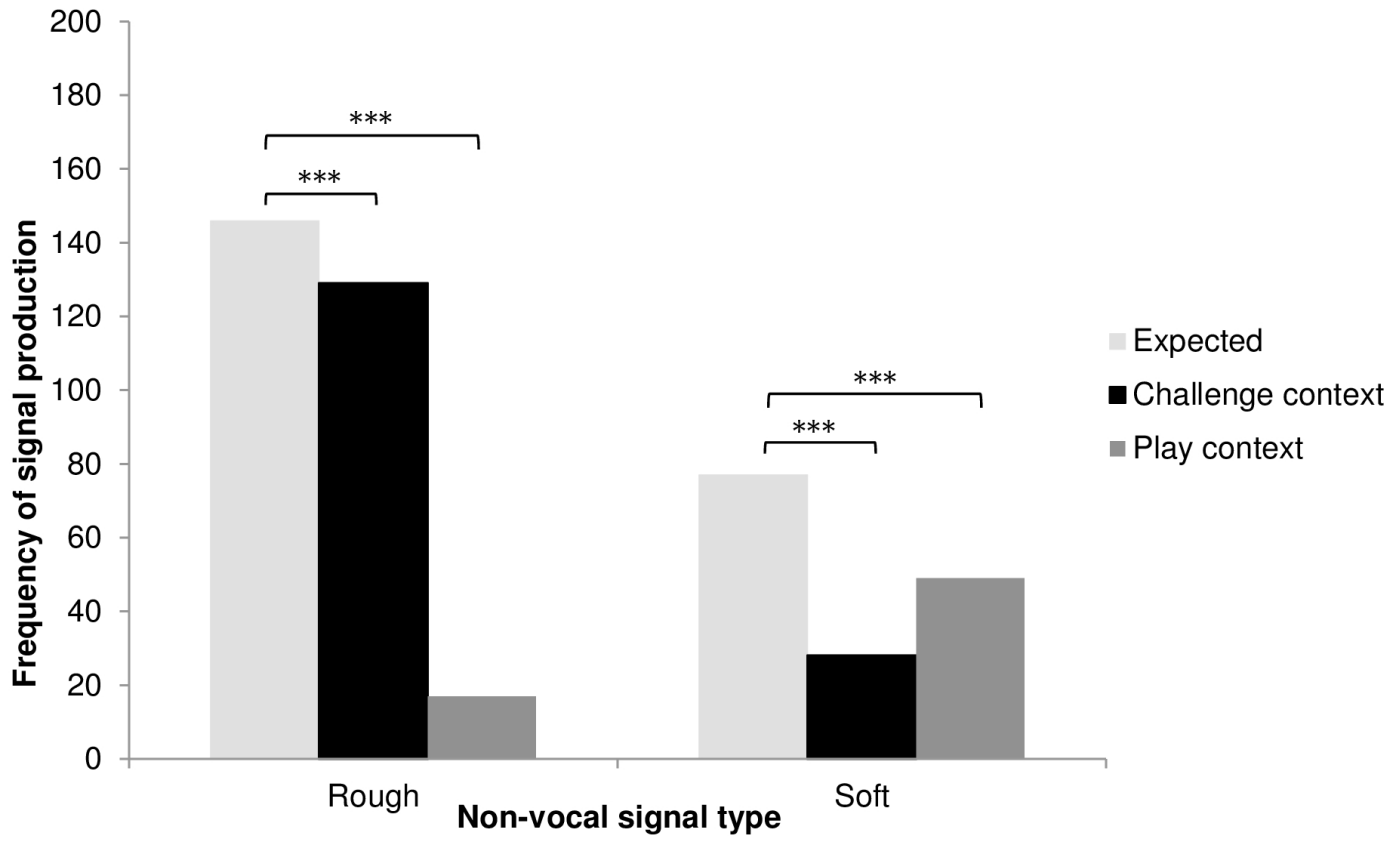
Frequency of production of rough and soft signals in multi-modal sequences with contest hoots. Light grey bars: expected values; black bars: observed values in the challenge context; dark grey: observed values in the play context. ***P<0.001

We then examined whether the putative goal of the signaller, i.e. to challenge or to play, was predicted by the structure of multi-modal sequences, i.e. by the type of signal, rough or soft, associated with contest hoots (see Table S3 for individual frequency of use of rough and soft signals). For the five males that used contest hoots in multi-modal sequences in both contexts, the individual ratios of rough and soft signals across contexts was not significantly different (Heterogeneity *G* = 6.21, *df* = 4, *P* = 0.18; Replicated G-test for goodness of fit, Table S3). When pooling individual data, we found that observed and expected frequencies of both rough and soft gestures were significantly different from each other (rough: *G* = 35.879, *df* = 1, *P*<0.0001; soft: *G* = 42.819, *P*<0.0001; goodness-of-fit test) with a higher proportion of rough signals in the challenge context and a higher proportion of soft signals in the play context (Figure 6), suggesting that the intended meaning was reinforced by the non-vocal elements of the multi-modal sequence. Nevertheless, in both contexts contest hoots were also given alone, suggesting that recipients might be faced with occasional ambiguities. However, at the time a male produced a contest hoot in the play context, play was usually already ongoing, suggesting that pragmatic cues (or context) helped the recipient disambiguate the signaller's intended meaning. If soft gestures are produced during play, they may serve to maintain an ongoing interaction, for example by reinforcing a playful mood and keeping the partner engaged in the activity.

Although the call was similar in both contexts, to either challenge a target individual or to play, multi-modal sequences differed in context-specific ways. While sequences in the play context were more likely to contain ‘soft’ gestures, sequences in the challenge context were more likely to contain ‘rough’ gestures. The acoustic analyses did not show significant structural differences between the calls in the two contexts, but of course it is always possible that more subtle acoustic features have remained unnoticed and that they influence the interpretation of calls. Nevertheless, our data are more consistent with the interpretation that these multi-modal sequences function to help convey the signaller's apparent goal, or as in the case of play, maintain an ongoing activity.

A systematic study of each signal's meaning is necessary to interpret how they are individually perceived and whether these multi-modal sequences are composed of redundant signals and serve to enhance the signal, or otherwise function to modulate or create new meaning [39]. Nevertheless, it is likely that multi-modal signals are perceived as a holistic message regardless of the composite parts [29], and form a single package that is treated and interpreted as a whole [78].

## Conclusions

Male bonobos produce acoustically distinct vocalisations, the ‘contest hoots’, in both socially untargeted and targeted ways. In the later case, males direct their calls to individuals of relatively high social status that have a propensity to react strongly. Contest hoots appear to function solely to challenge other group members, a non-aggressive way to assert social rank. Our results also suggest that, by demonstrating the ability to challenge high ranking individuals, contest hoots are a means to display the signaller's social status to a nearby audience and in this way possibly aid in reputation building. Somewhat surprisingly, multi-modal sequences were not more effective in eliciting reactions than contest hoots given alone, unless given by alpha males. However, multi-modal sequences were characterised by context-specificity of the gestural components, providing additional cues concerning the nature of the desired interaction. In sum, we have demonstrated that primate vocal behaviour, despite considerable acoustic inertia can be contextually flexible, socially directed, and deployed as part of context-specific, multi-modal combinations. We believe that these findings are relevant towards a more informed understanding of the evolution of human language.

## Supporting Information

Figure S1 **Some temporal and structural parameters measured on contest hoots**. Introductory phase duration (s)  =  e - a; transition onset (ΔHz)  =  frequency at (b) -frequency at (a); transition middle (ΔHz)  =  frequency at (d) - frequency at (b); transition offset (ΔHz)  =  frequency at (e) – frequency at (d); overall transition (ΔHz)  =  frequency at call end (e) - frequency at call beginning (a); distance to first stereotyped unit (s)  =  f - e; first stereotyped unit maximum pitch jump (ΔHz)  =  frequency at (g) - frequency at (f). Depicted is a time-frequency spectrogram of part of a contest hoot produced by Fizi.
(TIFF)

Table S1 **Group composition in terms of individual, sex, age class, social status, offspring and date of arrival at the sanctuary.**
(DOCX)

Table S2 **Individual frequencies of contest hoot production for each male signaller of groups 1 and 2 toward targets of different relative social rank.**
(DOCX)

Table S3 **Individual frequency of use of rough and soft signals (gestures and** ***body signals*** **) in multi-modal sequences with contest hoots, in the challenge and play contexts.**
(DOCX)

Methods S1 (DOCX)

Results S1 (DOCX)

References S1 (DOCX)

Video S1 (MOV)

Video S2 (MOV)

Video S3 (MOV)
