# Supplementary material for: Multi-Modal Use of a Socially Directed Call in Bonobos
Source: PLoS One. 2014 Jan 15;9(1):e84738. doi: 10.1371/journal.pone.0084738 (PMC3893130; doi:10.1371/journal.pone.0084738)
Supplement: Table S1 — Group composition in terms of individual, sex, age class, social status, offspring and date of arrival at the sanctuary. (DOCX) [file pone.0084738.s002.docx]

| Study group | Name | Sex | Age  class | Social status | Offspring  (Sex,YOB, social status) | Date of arrival |
| --- | --- | --- | --- | --- | --- | --- |
| 1 | Opala | F | A | H | Pole (M, 2006, L) | 1997 |
| 1 | Semendwa | F | A | α | Elikya (F, 2005, I); Makasi (M, 2010) | 1999 |
| 1 | Bandundu | F | A | H | Wangolo (M, 2008, L) | 1999 |
| 1 | Salonga | F | A | H | Kymia (F, 2009) | 2000 |
| 1 | Lisala | F | SA | H | Nyota (F, 2011) | 2003 |
| 1 | Katako | F | SA | L |  | 2007 |
| 1 | Waka | F | SA | L |  | 2008 |
| 1 | Manono | M | A | α |  | 1997 |
| 1 | Kikwit | M | A | I |  | 2000 |
| 1 | Fizi | M | SA | α |  | 2003 |
| 1 | Lomami | M | SA | I |  | 2005 |
| 1 | Api | M | SA | I |  | 2003 |
| 1 | Matadi | M | SA | I |  | 2002 |
| 1 | Mabali | M | SA | L |  | 2004 |
| 1 | Dilolo | M | SA | I |  | 2003 |
| 1 | Kasongo | M | SA | L |  | 2004 |
| 2 | Maya | F | A | α | Bisengo (M, 2005, I); Mayele (M, 2010) | 1995 |
| 2 | Kalina | F | A | H | Malaïka (F, 2007, L); Bolingo (M, 2011) | 1999 |
| 2 | Kisantu | F | A | H | Liyaka (F, 2010) | 2001 |
| 2 | Isiro | F | A | H |  | 2000 |
| 2 | Likasi | F | SA | I | Elonga (F, 2012) | 2003 |
| 2 | Muanda | F | SA | I |  | 2006 |
| 2 | Sake | F | SA | L |  | 2007 |
| 2 | Masisi | F | SA | L |  | 2008 |
| 2 | Keza | M | A | H |  | 2004 |
| 2 | Max | M | A | H |  | 2004 |
| 2 | Mbandaka | M | SA | α |  | 2005 |
| 2 | Bili | M | SA | L |  | 2003 |
| 2 | Ilebo | M | SA | I |  | 2005 |
| 2 | Yolo | M | SA | I |  | 2006 |
| 2 | Eleke | M | SA | L |  | 2007 |

**Table S1. Group composition in terms of individual, sex, age class, social status, offspring and date of arrival at the sanctuary.**

Sex; M: male, F: female. Age classes; A: adult, SA: subadult. Social status; H: high-ranking, I: intermediate-ranking, L: low-ranking (infants did not participate in the dominance analysis), α: dominant female and male individuals in the group (note that there are two α males marked for group 1, as there was a change in hierarchy during the study period).
