# Supplementary material for: Multi-Modal Use of a Socially Directed Call in Bonobos
Source: PLoS One. 2014 Jan 15;9(1):e84738. doi: 10.1371/journal.pone.0084738 (PMC3893130; doi:10.1371/journal.pone.0084738)
Supplement: Table S2 — Individual frequencies of contest hoot production for each male signaller of groups 1 and 2 toward targets of different relative social rank. (DOCX) [file pone.0084738.s003.docx]

**Table S2**. **Individual frequencies of contest hoot production for each male signaller of groups 1 and 2 toward targets of different relative social rank.**

|  |  |  |  | **Manono**  **(α)** | **Fizi**  **(α)** | **Api**  **(I)** | **Lomami**  **(I)** | **Matadi (I)** | **Dilolo (I)** | **Kikwit (I)** | **Mbandaka (α)** | **Keza (H)** | **Ilebo (H)** |
| --- | --- | --- | --- | --- | --- | --- | --- | --- | --- | --- | --- | --- | --- |
| **Status** | **Group** | **Sex** | **Target** |  |  |  |  |  |  |  |  |  |  |
| **HIGH** | 1 | F | **Semendwa α** | **21***** | 0 | 0 | 2 | 0 | 2 | 0 |  |  |  |
|  | 1 |  | Opala | 0 | 0 | 0 | 0 | 0 | 0 | 0 |  |  |  |
|  | 1 |  | Bandundu | **9***** | 0 | 0 | 4 | 0 | 6 | 0 |  |  |  |
|  | 1 |  | Salonga | 0 | **10***** | 0 | **21***** | 0 | 0 | 0 |  |  |  |
|  | 1 |  | Lisala | 2 | 0 | 0 | 9 | **3*** | 4 | 0 |  |  |  |
|  | 2 |  | **Maya α** |  |  |  |  |  |  |  | ***13****** | 3 | **13***** |
|  | 2 |  | Kalina |  |  |  |  |  |  |  | 4* | **22***** | 0 |
|  | 2 |  | Kisantu |  |  |  |  |  |  |  | 4* | **23***** | 1 |
|  | 2 |  | Isiro |  |  |  |  |  |  |  | 3 | **1** | 0 |
|  | 1 | M | Manono | 0 | **17***** | 7 | **14***** | 0 | 0 | 0 |  |  |  |
|  | 1 |  | Fizi | 1 | 0 | 0 | 1 | 0 | 0 | 0 |  |  |  |
|  | 2 |  | Mbandaka |  |  |  |  |  |  |  | 0 | **12***** | 0 |
|  | 2 |  | Keza |  |  |  |  |  |  |  | 0 | 0 | 0 |
|  | 2 |  | Max |  |  |  |  |  |  |  | 0 | **18***** | 1 |
| **INTER** | 1 | F | Elikya | 0 | 1 | 4 | 3 | 0 | 3 | 0 |  |  |  |
|  | 2 |  | Likasi |  |  |  |  |  |  |  | 0 | 1 | 0 |
|  | 2 |  | Muanda |  |  |  |  |  |  |  | 0 | 0 | 0 |
|  | 1 | M | Lomami | **24***** | 7 | **47***** | 0 | 0 | 1 | **3** |  |  |  |
|  | 1 |  | Api | **16***** | 2 | 0 | **47***** | ***7****** | **9***** | **4** |  |  |  |
|  | 1 |  | Matadi | 0 | 0 | ***65****** | ***25****** | 0 | 0 | 0 |  |  |  |
|  | 1 |  | Kikwit | 0 | 0 | 3 | 5 | 0 | 0 | 0 |  |  |  |
|  | 1 |  | Dilolo | 0 | 0 | 7 | 1 | 0 | 0 | **5** |  |  |  |
|  | 2 |  | Ilebo |  |  |  |  |  |  |  | 0 | 0 | 0 |
|  | 2 |  | Yolo |  |  |  |  |  |  |  | 0 | 0 | 3 |
|  | 2 |  | Bisengo |  |  |  |  |  |  |  | 0 | 1 | 0 |
| **LOW** | 1 | F | Katako | 0 | 1 | 0 | 0 | 0 | 0 | 0 |  |  |  |
|  | 1 |  | Waka | 0 | 0 | 0 | 0 | 0 | 0 | 0 |  |  |  |
|  | 2 |  | Sake |  |  |  |  |  |  |  | 0 | 3 | 0 |
|  | 2 |  | Malaika |  |  |  |  |  |  |  | 0 | 1 | 0 |
|  | 2 |  | Masisi |  |  |  |  |  |  |  | 0 | 0 | 0 |
|  | 1 | M | Mabali | 0 | 0 | 0 | 0 | 2 | ***20****** | 0 |  |  |  |
|  | 1 |  | Kasongo | 0 | 0 | 0 | 1 | 0 | 2 | 1 |  |  |  |
|  | 1 |  | Pole | 0 | 0 | 3 | 4 | 1 | 0 | 0 |  |  |  |
|  | 1 |  | Wangolo | 0 | 0 | 0 | 0 | 0 | 0 | 0 |  |  |  |
|  | 2 |  | Bili |  |  |  |  |  |  |  | 0 | 0 | 0 |
|  | 2 |  | Eleke |  |  |  |  |  |  |  | 0 | 0 | 0 |
|  |  |  |  | **73** | **38** | **136** | **137** | **13** | **47** | **13** | **24** | **85** | **18** |
| G |  |  |  | 299.33 | 195.68 | 827.51 | 384.02 | 83.92 | 176.71 | 65.46 | 133.5 | 233.28 | 143.8 |
| *df* |  |  |  | 19 | 19 | 19 | 19 | 19 | 19 | 19 | 17 | 17 | 17 |
| *P* |  |  |  | <0.001 | <0.001 | <0.001 | <0.001 | <0.001 | <0.001 | <0.001 | <0.001 | <0.001 | <0.001 |

Sex; M: male, F: female. Social status; α: alpha, H: high-ranking, I: intermediate-ranking. Bold caption: production of contest hoots (uni- and multi-modal) to preferred targets (binomial test: *** *P*<0.001, ***P*<0.01, **P*<0.05). Italic caption: frequency of production significantly higher in the play context (exact binomial test: *P*<0.001). At the bottom of the table are represented the results of the Replicated goodness of fit test for individual distribution of preferred targets.
