# Supplementary material for: Multi-Modal Use of a Socially Directed Call in Bonobos
Source: PLoS One. 2014 Jan 15;9(1):e84738. doi: 10.1371/journal.pone.0084738 (PMC3893130; doi:10.1371/journal.pone.0084738)
Supplement: Table S3 — Individual frequency of use of rough and soft signals (gestures and body signals ) in multi-modal sequences with contest hoots, in the challenge and play contexts. (DOCX) [file pone.0084738.s004.docx]

| **CHALLENGE CONTEXT** | | | | | | | | | | |
| --- | --- | --- | --- | --- | --- | --- | --- | --- | --- | --- |
|  | **Api** | **Manono** | **Lomami** | **Fizi** | **Dilolo** | **Matadi** | **Kikwit** | **Keza** | **Ilebo** | **Mbandaka** |
| **Rough signals** |  |  |  |  |  |  |  |  |  |  |
| Arm swing/with object | 1 | 12 | 10 | 4 | 6 | 1 |  | 14 | 16 |  |
| Flap/with object | 6 | 9 | 10 | 1 | 3 |  | 1 | 7 | 3 | 1 |
| Hit with object | 1 |  |  |  |  |  |  |  | 1 |  |
| Hit ground with object |  | 2 |  |  | 1 |  |  |  |  |  |
| Kick |  |  | 1 | 1 | 1 |  |  |  |  |  |
| Object shake |  | 1 | 1 |  | 1 |  |  | 2 |  |  |
| Push | 4 |  | 2 |  |  |  |  |  | 1 |  |
| Rap object |  |  |  |  |  |  |  | 18 |  |  |
| Rhythmic stomp/stomp |  | 16 | 1 |  |  | 1 |  |  | 4 |  |
| Slap other | 3 | 5 | 6 | 3 | 2 |  | 2 | 2 |  | 2 |
| Slap object |  |  | 1 | 2 |  |  |  | 2 |  |  |
| Throw object | 6 | 21 | 1 | 6 | 1 | 1 | 1 | 4 |  | 1 |
| *Bipedal swagger* |  | 10 | 4 | 2 | 1 |  |  | 3 |  | 1 |
| *Object dragging* | 20 | 7 | 1 | 3 | 4 | 1 | 1 | 11 |  | 1 |
| *Push object* |  | 8 |  | 1 |  | 1 |  | 13 |  |  |
| *Stiff trot* |  |  |  |  |  |  |  | 3 |  |  |
| **Total** | **41** | **91** | **38** | **23** | **20** | **5** | **5** | **79** | **25** | **6** |
| **Soft signals** |  |  |  |  |  |  |  |  |  |  |
| Arm raise/with object | 3 |  | 1 |  | 1 |  |  | 1 | 1 |  |
| Hand wave off | 2 |  |  |  | 1 |  |  |  |  |  |
| Hand-down reach | 1 |  |  |  | 1 |  |  |  |  |  |
| Hand-side reach |  |  |  |  | 3 |  |  |  |  |  |
| Stretch over | 1 |  | 1 | 2 |  |  | 3 |  |  | 3 |
| Touch |  | 2 | 1 |  |  |  |  |  |  |  |
| Wrist shake |  |  |  |  | 1 |  |  |  |  |  |
| *Bipedal present* |  |  |  |  |  |  |  | 2 |  |  |
| *Concave back present* |  |  | 5 | 7 | 1 |  |  |  | 1 |  |
| *Rump present* | 2 |  |  |  |  |  |  |  |  |  |
| **Total** | **9** | **2** | **8** | **9** | **8** | **0** | **3** | **3** | **2** | **3** |
| **PLAY CONTEXT** | | | | | | | | | | |
|  | **Api** | **Manono** | **Lomami** | **Fizi** | **Dilolo** | **Matadi** | **Kikwit** | **Keza** | **Ilebo** | **Mbandaka** |
| **Rough signals** |  | | | | | | | | | |
| Arm swing/with object | 2 |  | 1 |  | 1 |  |  |  | 5 |  |
| Kick |  |  | 1 |  | 1 |  |  |  |  |  |
| Object shake |  |  |  |  | 1 |  |  |  |  |  |
| Push | 1 |  |  |  |  |  |  |  |  |  |
| Slap other | 1 |  |  |  | 1 |  |  |  |  |  |
| Bipedal swagger |  |  | 1 |  |  |  |  |  |  |  |
| Object dragging |  |  |  |  | 1 |  |  |  |  |  |
| **Total** | **4** | **0** | **3** | **0** | **5** | **0** | **0** | **0** | **5** | **0** |
| **Soft signals** |  | | | | | | | | | |
| Arm raise/with object | 1 |  | 1 |  |  |  |  |  |  |  |
| Grab/grab-pull | 1 |  | 11 |  |  |  |  |  |  |  |
| Hand-down reach | 1 |  |  |  |  |  |  |  |  |  |
| Hand-side reach | 9 |  |  |  |  |  |  |  |  |  |
| Hand-up reach | 16 |  |  |  |  | 1 |  |  |  |  |
| Touch |  |  | 5 |  |  |  |  |  | 1 |  |
| Wrist shake | 1 |  |  |  |  |  |  |  |  |  |
| *Rump present* |  |  | 1 |  |  |  |  |  |  |  |
| **Total** | **29** | **0** | **18** | **0** | **0** | **1** | **0** | **0** | **1** | **0** |

**Table S3. Individual frequency of use of rough and soft signals (gestures and *body signals*) in multimodal sequences with contest hoots, in the challenge and play contexts**
