# Supplementary material for: Multi-Modal Use of a Socially Directed Call in Bonobos
Source: PLoS One. 2014 Jan 15;9(1):e84738. doi: 10.1371/journal.pone.0084738 (PMC3893130; doi:10.1371/journal.pone.0084738)
Supplement: Methods S1 — (DOCX) [file pone.0084738.s005.docx]

**Supporting methods**

*Social history*

Most individuals were former victims of the pet trade, having arrived at the sanctuary as young orphans after their mothers had been killed by poachers. Previous studies have not found any behavioural or cognitive abnormalities in these animals, nor measurable signs of psychological distress, due to their translocation to the sanctuary [1], but a recent study demonstrated that mother-reared infants were more responsive to distress in others than orphans, thus highlighting the importance of rearing in emotional development [2]. From 1995 to 2001, the first orphans lived in one nursery group. In 2001, a first adult group was formed in addition to this nursery group, followed by a second adult group in 2004. Newly arriving individuals spend at least two years in a nursery group before being transferred to an adult group. Transfers between the adult groups are also common and groups are separated by a shared fence. As a result, all adult and subadult individuals know each other, have regular visual contact, and have at one time lived in the same group.

*Statistical analyses*

When data were not appropriate for parametric statistics, transformations were performed. *Z* values > 1.96 and < -1.96 were considered to be positively or negatively skewed. In the case of positive skew a transformation of √(*X*) or √(*X* + 101) was applied. In the case of negative skew we applied the transformation: √((*X* max + 1) - *X*), where *X* max = the highest value within the data set. If these transformations continued to generate *Z* values outside the appropriate bounds we used non-parametric analyses. If homogeneity of variance was insufficient, following a Levene’s test, we used alternatives that did not require homogenous data.

*Acoustic analysis*

The acoustic analyses were based on the following 18 parameters: (1) total call duration (s); (2) introductory phase presence or absence (3) introductory phase duration (s); (4) introductory phase transition onset (∆Hz): frequency of maximum energy at beginning of call, minus frequency of maximum energy at call second quarter; (5) introductory phase transition middle (∆Hz): frequency of maximum energy at end of third quarter, minus frequency of maximum energy at beginning of second quarter; (6) introductory phase offset (∆Hz): frequency of maximum energy at end of call, minus frequency of maximum energy at end of third quarter; (7) introductory phase pitch jump (∆Hz): frequency of maximum energy in the call, minus frequency of minimal energy in the call; (8) distance to first stereotyped unit (∆s): time at beginning of first stereotyped unit, minus time at end of introductory phase; (9) first stereotyped unit presence or absence; (10) first stereotyped unit duration (s); (11) first stereotyped unit pitch jump (∆Hz); (12) distance to next unit of escalation (∆s); (13) escalation presence or absence; (14) escalation duration (s); (15) number of units in escalation; (16) minimum distance between two units during escalation (∆s); (17) maximum pitch-jump during escalation (∆Hz); (18) let-down phase presence or absence (fig. S1).

We screened the data for outliers by producing standardized Z scores, rejecting calls with a Z score greater than 3.29 in one or more parameters [3]. We then regressed all parameters to check for co-linearity and removed parameters with a variance inflation factor greater than 10 [3].
