# Supplementary material for: Multi-Modal Use of a Socially Directed Call in Bonobos
Source: PLoS One. 2014 Jan 15;9(1):e84738. doi: 10.1371/journal.pone.0084738 (PMC3893130; doi:10.1371/journal.pone.0084738)
Supplement: Results S1 — (DOCX) [file pone.0084738.s006.docx]

**Supporting results**

*Multi-modal sequence composition*

Contest hoots could be given alone (means ± SE: 49.6±16.8%, *N*=10 males) or as part of multi-modal sequences (means ± SE: 50.4±16.8%, *N*=10 males). Multi-modal sequences were composed of an average of 2.73 (±1.1) signals (including contest hoots). The sequences were significantly more often initiated with a contest hoot compared to a gesture or body signal (65.0±21.0% of cases, *N*=10 males; repeated measures ANOVA with Greenhouse-Geisser correction, *F*=33.821, *df* =1.4, *P*<0.0001). The mean latency between the beginning of a contest hoot and the onset of a non-vocal signal was 2.7s (± 2.9s), but in the majority of cases (74.0%) the latency was ≤ 1.0s. Contest hoots were usually produced throughout the entire multi-modal sequence ending on average 1.0s after the last non-vocal signal.

The temporal organisation of multi-modal sequences described here was similar to what has been described by Pollick et al. [4] for captive bonobos and by van Hooff [5] for chimpanzees, where facial or vocal signals tended to occur prior to gestures. Regarding the structural organisation of multi-modal sequences, Partan & Marler [6] argued that the choice of the first signal might have an effect on the recipient responsiveness, i.e. if the first signal is audible it might serve to attract attention and influence the success of the entire communication event. Alternatively, Pollick et al. [4] argue that vocalisations are under less voluntary control than gestures, so that the primacy of vocalisations in multi-modal sequences can be explained as a mere by-product of differences in signal control.

*Distance to recipient*

Although male bonobos frequently produced contest hoots in a socially untargeted ways, they also directed contest hoots, alone or in combination with other signals, towards specific individuals. In the targeted cases, the distance between signaller and recipient was always very short, around 2-3 meters, when signalling started with no significant difference between modalities (uni-modal: 2.4±0.7m, multi-modal: 2.3 ±0.7m, means ± SE; *N*=10, *t*-test, two-tailed: *t*=0.448, *df*=9, *P*=0.665).
