# Supplementary material for: Multi-Modal Use of a Socially Directed Call in Bonobos
Source: PLoS One. 2014 Jan 15;9(1):e84738. doi: 10.1371/journal.pone.0084738 (PMC3893130; doi:10.1371/journal.pone.0084738)
Supplement: References S1 — (DOCX) [file pone.0084738.s007.docx]

**Supporting references**

1. Wobber V, Hare B (2011) Psychological health of orphan bonobos and chimpanzees in African sanctuaries. PLoS ONE 6(6): e17147. doi:10.1371/ journal.pone.0017147

2. Clay Z, de Waal F (2013) Bonobos respond to distress in others: Consolation across the age spectrum. PLoS ONE 8(1): e55206.doi:10.1371/journal.pone.0055206

3. Tabachnick BG, Fidell LS (2001) Using multivariate statistics, 4th edn. Boston: Allyn & Bacon.

4. Pollick AS, Jeneson A, de Waal FB (2008) Gestures and multimodal signaling in bonobos. In Furuichi T, Thompson J, editors. The Bonobos: Behavior, Ecology, and Conservation. Springer. pp. 75-94.

5. Van Hooff JARAM (1973) Structural analysis of the social behaviour of a semi-captive group of chimpanzees. In von Cranach M, Vine I. editors. Social Communication and Movement, Studies of Interaction and Expression in Man and Chimpanzee. London & New York: Academic Press. pp. 75-162.

6. Partan SR, Marler P (2005) Issues in the classification of multimodal communication signals. Am Nat 166: 231–245.
